# Ethyl 2-{4-[(1,5-dibenzyl-2,4-dioxo-2,3,4,5-tetra­hydro-1*H*-1,5-benzo­diazepin-3-yl)meth­yl]-1*H*-1,2,3-triazol-1-yl}acetate

**DOI:** 10.1107/S1600536809052696

**Published:** 2009-12-12

**Authors:** Hind Jabli, Y. Kandri Rodi, Sonia Ladeira, El Mokhtar Essassi, Seik Weng Ng

**Affiliations:** a Laboratoire de Chimie Organique Appliquée, Faculté des Sciences et Techniques, Université Sidi Mohamed Ben Abdallah, Fés, Morocco; b Service de Diffraction X, Laboratoire de Chimie de Coordination, 205 route de Narbonne, 31077 Toulouse Cedex 04, France; c Laboratoire de Chimie Organique Hétérocyclique, Pôle de compétences Pharmacochimie, Université Mohammed V-Agdal, BP 1014 Avenue Ibn Batout, Rabat, Morocco; d Department of Chemistry, University of Malaya, 50603 Kuala Lumpur, Malaysia

## Abstract

The reaction of 1,5-dibenzyl-3-propargyl-1,5-benzodiazepine-2,4-dione with ethyl azido­acetate in the presence of copper sulfate pentahydrate and sodium ascorbate leads to the formation of the title regioisomer, C_30_H_29_N_5_O_4_, which features a phenyl­ene ring fused with a seven-membered diazepinyl ring. The latter ring adopts a boat conformation (with the methyl­triazolylacetate-bearing C atom as the prow and the fused-ring C atoms as the stern). The benzyl groups connected to the diazepinyl ring jprotrude from the sides; the methyl­triazolylacetate substituent occupies an axial position.

## Related literature

For the crystal structure of the parent compound, benzodiazepin-2,4-dione, see: Négrier *et al.* (2006 ▶). For the crystal structure of 1,5-dibenzyl-3-propargyl-1,5-benzodiazepine-2,4-dione, see: Jabli *et al.* (2009 ▶).
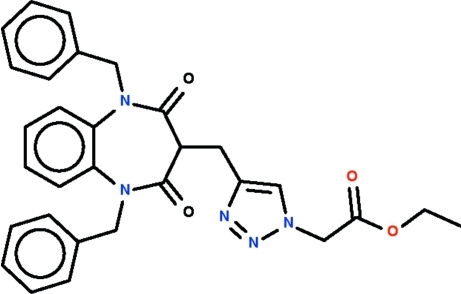


## Experimental

### 

#### Crystal data

C_30_H_29_N_5_O_4_
*M* *_r_* = 523.58
Monoclinic, 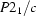

*a* = 14.2015 (5) Å
*b* = 10.9337 (4) Å
*c* = 17.9368 (6) Å
β = 95.699 (2)°
*V* = 2771.37 (17) Å^3^
*Z* = 4
Mo *K*α radiation
μ = 0.09 mm^−1^
*T* = 243 K
0.30 × 0.22 × 0.08 mm

#### Data collection

Bruker APEXII diffractometer
29097 measured reflections
6377 independent reflections
2690 reflections with *I* > 2σ(*I*)
*R*_int_ = 0.079
Standard reflections: 0

#### Refinement

*R*[*F* ^2^ > 2σ(*F* ^2^)] = 0.058
*wR*(*F* ^2^) = 0.174
*S* = 1.00
6377 reflections
352 parameters
1 restraint
H-atom parameters constrained
Δρ_max_ = 0.48 e Å^−3^
Δρ_min_ = −0.22 e Å^−3^

### 

Data collection: *APEX2* (Bruker, 2005 ▶); cell refinement: *SAINT* (Bruker, 2005 ▶); data reduction: *SAINT*; program(s) used to solve structure: *SHELXS97* (Sheldrick, 2008 ▶); program(s) used to refine structure: *SHELXL97* (Sheldrick, 2008 ▶); molecular graphics: *X-SEED* (Barbour, 2001 ▶); software used to prepare material for publication: *publCIF* (Westrip, 2009 ▶).

## Supplementary Material

Crystal structure: contains datablocks global, I. DOI: 10.1107/S1600536809052696/sj2708sup1.cif

Structure factors: contains datablocks I. DOI: 10.1107/S1600536809052696/sj2708Isup2.hkl

Additional supplementary materials:  crystallographic information; 3D view; checkCIF report

## Figures and Tables

**Fig. 1.**
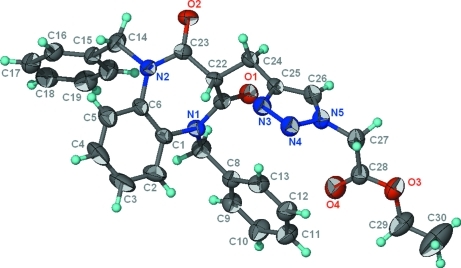
Thermal ellipsoid plot (Barbour, 2001) of C30H29N5O4 at the 70% probability level; hydrogen atoms are drawn as arbitrary radius.

## supplementary crystallographic information

### Experimental

To a solution 1,5-dibenzyl-1,5-benzodiazepine-2,4-dione (1 mmol) *t*-butyl alcohol/water (1/2, 8 ml) was added copper sulfate pentahydrate (1 mmol), sodium ascorbate (2 mmol) and ethyl azidoacetate (5 mmol). Stirring was continued for 8 h. The solution was diluted with water (20 ml) and the organic compound extracted with ethyl acetate (2 *x* 20 ml). The extracts were washed with brine and dried over sodium sulfate. The compound was recrystallized from ether to give colorless crystals.

### Refinement

Carbon-bound H-atoms were placed in calculated positions (C—H 0.94 to 0.98 Å) and were included in the refinement in the riding model approximation, with *U*(H) set to 1.2–1.5*U*(C).

The carbon-carbon distance in the ethyl end of the molecule was tightly restrained to 1.540±0.005 Å. Attempts to model this unit as being disordered over two sites required a large number of restraints.

### Crystal data

**Table:**

C_30_H_29_N_5_O_4_	*F*(000) = 1104
*M**_r_* = 523.58	*D*_x_ = 1.255 Mg m^−^^3^
Monoclinic, *P*2_1_/*c*	Mo *K*α radiation, λ = 0.71073 Å
Hall symbol: -P 2ybc	Cell parameters from 2371 reflections
*a* = 14.2015 (5) Å	θ = 2.2–18.1°
*b* = 10.9337 (4) Å	µ = 0.09 mm^−^^1^
*c* = 17.9368 (6) Å	*T* = 243 K
β = 95.699 (2)°	Block, colorless
*V* = 2771.37 (17) Å^3^	0.30 × 0.22 × 0.08 mm
*Z* = 4	

### Data collection

**Table:**

Bruker APEXII diffractometer	2690 reflections with *I* > 2σ(*I*)
Radiation source: fine-focus sealed tube	*R*_int_ = 0.079
graphite	θ_max_ = 27.5°, θ_min_ = 1.4°
φ and ω scans	*h* = −15→18
29097 measured reflections	*k* = −11→14
6377 independent reflections	*l* = −23→23

### Refinement

**Table:**

Refinement on *F*^2^	Primary atom site location: structure-invariant direct methods
Least-squares matrix: full	Secondary atom site location: difference Fourier map
*R*[*F*^2^ > 2σ(*F*^2^)] = 0.058	Hydrogen site location: inferred from neighbouring sites
*wR*(*F*^2^) = 0.174	H-atom parameters constrained
*S* = 1.00	*w* = 1/[σ^2^(*F*_o_^2^) + (0.0719*P*)^2^ + 0.0345*P*] where *P* = (*F*_o_^2^ + 2*F*_c_^2^)/3
6377 reflections	(Δ/σ)_max_ = 0.001
352 parameters	Δρ_max_ = 0.48 e Å^−^^3^
1 restraint	Δρ_min_ = −0.22 e Å^−^^3^

### Fractional atomic coordinates and isotropic or equivalent isotropic displacement parameters (Å^2^)

**Table:**

	*x*	*y*	*z*	*U*_iso_*/*U*_eq_	
O1	0.37697 (14)	1.09779 (17)	0.39010 (11)	0.0567 (6)	
O2	0.11543 (14)	1.08866 (18)	0.41866 (11)	0.0600 (6)	
O3	0.70046 (15)	0.63752 (18)	0.54560 (12)	0.0665 (6)	
O4	0.60809 (16)	0.7150 (2)	0.44777 (13)	0.0844 (8)	
N1	0.31956 (15)	1.0200 (2)	0.27730 (12)	0.0482 (6)	
N2	0.11754 (15)	1.0312 (2)	0.29647 (12)	0.0483 (6)	
N3	0.31787 (17)	0.7234 (2)	0.46224 (13)	0.0557 (7)	
N4	0.38706 (18)	0.6481 (2)	0.48645 (13)	0.0576 (7)	
N5	0.45203 (16)	0.7143 (2)	0.52910 (12)	0.0484 (6)	
C1	0.2513 (2)	0.9476 (3)	0.23315 (14)	0.0471 (7)	
C2	0.2827 (3)	0.8723 (3)	0.17821 (18)	0.0732 (10)	
H2	0.3478	0.8658	0.1735	0.088*	
C3	0.2187 (3)	0.8067 (4)	0.1304 (2)	0.0931 (13)	
H3	0.2404	0.7552	0.0938	0.112*	
C4	0.1223 (3)	0.8172 (3)	0.13675 (19)	0.0830 (11)	
H4	0.0786	0.7753	0.1031	0.100*	
C5	0.0908 (2)	0.8889 (3)	0.19218 (16)	0.0630 (9)	
H5	0.0256	0.8934	0.1971	0.076*	
C6	0.1540 (2)	0.9551 (2)	0.24119 (14)	0.0455 (7)	
C7	0.38849 (19)	1.0938 (3)	0.23979 (16)	0.0544 (8)	
H7A	0.3887	1.1768	0.2604	0.065*	
H7B	0.3658	1.0996	0.1865	0.065*	
C8	0.49003 (19)	1.0480 (2)	0.24595 (15)	0.0459 (7)	
C9	0.5521 (2)	1.1034 (3)	0.20078 (16)	0.0561 (8)	
H9	0.5294	1.1643	0.1666	0.067*	
C10	0.6470 (2)	1.0702 (3)	0.2054 (2)	0.0703 (10)	
H10	0.6875	1.1073	0.1737	0.084*	
C11	0.6819 (2)	0.9829 (3)	0.25645 (19)	0.0705 (9)	
H11	0.7464	0.9618	0.2604	0.085*	
C12	0.6217 (2)	0.9267 (3)	0.30160 (18)	0.0628 (9)	
H12	0.6451	0.8667	0.3362	0.075*	
C13	0.5257 (2)	0.9588 (3)	0.29596 (16)	0.0558 (8)	
H13	0.4849	0.9194	0.3265	0.067*	
C14	0.0279 (2)	1.0971 (3)	0.27638 (17)	0.0630 (9)	
H14A	0.0149	1.1499	0.3183	0.076*	
H14B	−0.0237	1.0375	0.2688	0.076*	
C15	0.0286 (2)	1.1742 (3)	0.20694 (15)	0.0498 (7)	
C16	−0.0512 (2)	1.1786 (3)	0.15552 (17)	0.0598 (8)	
H16	−0.1045	1.1314	0.1636	0.072*	
C17	−0.0530 (3)	1.2522 (3)	0.09205 (19)	0.0702 (10)	
H17	−0.1071	1.2538	0.0574	0.084*	
C18	0.0242 (3)	1.3224 (3)	0.08008 (19)	0.0728 (10)	
H18	0.0227	1.3720	0.0372	0.087*	
C19	0.1046 (2)	1.3204 (3)	0.1311 (2)	0.0711 (9)	
H19	0.1572	1.3689	0.1231	0.085*	
C20	0.1064 (2)	1.2457 (3)	0.19446 (18)	0.0637 (9)	
H20	0.1607	1.2438	0.2289	0.076*	
C21	0.32027 (19)	1.0305 (2)	0.35331 (16)	0.0449 (7)	
C22	0.24439 (18)	0.9589 (2)	0.38906 (14)	0.0408 (6)	
H22	0.2373	0.8774	0.3652	0.049*	
C23	0.15274 (19)	1.0309 (2)	0.37117 (16)	0.0456 (7)	
C24	0.27037 (19)	0.9424 (2)	0.47331 (14)	0.0475 (7)	
H24A	0.2986	1.0181	0.4945	0.057*	
H24B	0.2129	0.9260	0.4977	0.057*	
C25	0.33895 (19)	0.8388 (2)	0.48895 (14)	0.0424 (7)	
C26	0.4240 (2)	0.8323 (2)	0.53126 (15)	0.0493 (7)	
H26	0.4567	0.8969	0.5568	0.059*	
C27	0.5400 (2)	0.6571 (3)	0.56053 (16)	0.0526 (8)	
H27A	0.5294	0.5695	0.5674	0.063*	
H27B	0.5594	0.6927	0.6098	0.063*	
C28	0.6185 (2)	0.6746 (3)	0.51023 (19)	0.0557 (8)	
C29	0.7809 (2)	0.6436 (4)	0.5009 (2)	0.0954 (13)	
H29A	0.7979	0.7291	0.4928	0.114*	
H29B	0.7645	0.6052	0.4520	0.114*	
C30	0.8613 (3)	0.5788 (4)	0.5417 (3)	0.155 (2)	
H30A	0.9154	0.5819	0.5127	0.232*	
H30B	0.8441	0.4942	0.5493	0.232*	
H30C	0.8774	0.6177	0.5899	0.232*	

### Atomic displacement parameters (Å^2^)

**Table:**

	*U*^11^	*U*^22^	*U*^33^	*U*^12^	*U*^13^	*U*^23^
O1	0.0539 (13)	0.0544 (12)	0.0612 (13)	−0.0108 (10)	0.0030 (11)	−0.0064 (10)
O2	0.0620 (14)	0.0655 (13)	0.0542 (13)	0.0135 (11)	0.0145 (11)	−0.0095 (11)
O3	0.0555 (14)	0.0591 (14)	0.0836 (16)	0.0036 (11)	−0.0003 (13)	0.0102 (11)
O4	0.0740 (17)	0.120 (2)	0.0600 (15)	0.0123 (14)	0.0093 (13)	0.0211 (14)
N1	0.0452 (14)	0.0583 (15)	0.0424 (14)	−0.0054 (12)	0.0108 (12)	0.0026 (11)
N2	0.0456 (14)	0.0554 (14)	0.0442 (14)	0.0092 (11)	0.0058 (11)	0.0036 (11)
N3	0.0560 (16)	0.0506 (15)	0.0588 (16)	0.0016 (13)	−0.0017 (13)	−0.0098 (12)
N4	0.0586 (17)	0.0468 (14)	0.0656 (17)	−0.0035 (13)	−0.0027 (14)	−0.0102 (13)
N5	0.0538 (15)	0.0400 (13)	0.0500 (14)	−0.0046 (12)	−0.0013 (12)	0.0011 (11)
C1	0.0504 (19)	0.0582 (18)	0.0341 (15)	−0.0024 (15)	0.0103 (14)	−0.0017 (13)
C2	0.074 (2)	0.092 (3)	0.059 (2)	−0.005 (2)	0.0277 (19)	−0.0179 (19)
C3	0.106 (3)	0.117 (3)	0.060 (2)	−0.021 (3)	0.029 (2)	−0.041 (2)
C4	0.096 (3)	0.107 (3)	0.047 (2)	−0.036 (2)	0.010 (2)	−0.022 (2)
C5	0.063 (2)	0.080 (2)	0.0463 (19)	−0.0135 (18)	0.0049 (17)	0.0028 (17)
C6	0.0501 (19)	0.0516 (17)	0.0346 (15)	−0.0020 (14)	0.0037 (14)	0.0011 (13)
C7	0.0527 (19)	0.0597 (19)	0.0523 (18)	−0.0017 (15)	0.0127 (15)	0.0151 (14)
C8	0.0487 (18)	0.0422 (16)	0.0476 (17)	−0.0058 (14)	0.0077 (14)	−0.0022 (14)
C9	0.055 (2)	0.0580 (19)	0.0560 (19)	−0.0057 (16)	0.0088 (16)	0.0116 (15)
C10	0.053 (2)	0.078 (2)	0.082 (2)	−0.0051 (18)	0.0116 (19)	0.0160 (19)
C11	0.055 (2)	0.077 (2)	0.081 (2)	0.0072 (18)	0.012 (2)	0.007 (2)
C12	0.068 (2)	0.0550 (19)	0.066 (2)	0.0146 (17)	0.0066 (18)	0.0096 (16)
C13	0.064 (2)	0.0494 (18)	0.0564 (19)	0.0017 (16)	0.0162 (16)	0.0082 (15)
C14	0.0445 (19)	0.079 (2)	0.066 (2)	0.0117 (16)	0.0066 (16)	0.0145 (17)
C15	0.0445 (18)	0.0548 (18)	0.0497 (18)	0.0076 (15)	0.0027 (15)	0.0049 (14)
C16	0.051 (2)	0.065 (2)	0.064 (2)	0.0076 (16)	0.0083 (17)	0.0019 (17)
C17	0.067 (2)	0.081 (3)	0.061 (2)	0.020 (2)	0.0015 (19)	0.0083 (18)
C18	0.095 (3)	0.067 (2)	0.058 (2)	0.022 (2)	0.016 (2)	0.0151 (17)
C19	0.071 (2)	0.067 (2)	0.078 (2)	−0.0035 (18)	0.019 (2)	0.0073 (19)
C20	0.055 (2)	0.069 (2)	0.067 (2)	−0.0023 (17)	0.0032 (17)	0.0048 (17)
C21	0.0426 (17)	0.0426 (16)	0.0500 (18)	0.0038 (14)	0.0067 (15)	−0.0001 (14)
C22	0.0447 (16)	0.0393 (15)	0.0391 (15)	0.0020 (13)	0.0082 (13)	−0.0030 (12)
C23	0.0482 (18)	0.0458 (16)	0.0438 (17)	−0.0001 (14)	0.0102 (15)	−0.0004 (13)
C24	0.0536 (18)	0.0520 (17)	0.0372 (15)	0.0009 (14)	0.0068 (14)	−0.0028 (13)
C25	0.0478 (18)	0.0437 (16)	0.0359 (15)	−0.0039 (14)	0.0048 (14)	−0.0012 (12)
C26	0.059 (2)	0.0362 (16)	0.0519 (18)	−0.0065 (14)	0.0006 (16)	−0.0020 (13)
C27	0.059 (2)	0.0411 (16)	0.0550 (18)	0.0026 (14)	−0.0058 (16)	0.0086 (14)
C28	0.056 (2)	0.0488 (18)	0.061 (2)	0.0044 (15)	−0.0026 (18)	0.0041 (15)
C29	0.060 (2)	0.110 (3)	0.118 (3)	0.012 (2)	0.017 (3)	0.013 (3)
C30	0.089 (3)	0.141 (5)	0.235 (7)	0.038 (3)	0.021 (4)	0.056 (4)

### Geometric parameters (Å, °)

**Table:**

O1—C21	1.233 (3)	C11—H11	0.9400
O2—C23	1.223 (3)	C12—C13	1.402 (4)
O3—C28	1.333 (3)	C12—H12	0.9400
O3—C29	1.461 (4)	C13—H13	0.9400
O4—C28	1.200 (3)	C14—C15	1.504 (4)
N1—C21	1.367 (3)	C14—H14A	0.9800
N1—C1	1.429 (3)	C14—H14B	0.9800
N1—C7	1.481 (3)	C15—C16	1.388 (4)
N2—C23	1.383 (3)	C15—C20	1.390 (4)
N2—C6	1.430 (3)	C16—C17	1.392 (4)
N2—C14	1.477 (3)	C16—H16	0.9400
N3—N4	1.322 (3)	C17—C18	1.372 (5)
N3—C25	1.371 (3)	C17—H17	0.9400
N4—N5	1.350 (3)	C18—C19	1.391 (5)
N5—C26	1.351 (3)	C18—H18	0.9400
N5—C27	1.460 (3)	C19—C20	1.397 (4)
C1—C2	1.391 (4)	C19—H19	0.9400
C1—C6	1.405 (4)	C20—H20	0.9400
C2—C3	1.385 (5)	C21—C22	1.524 (4)
C2—H2	0.9400	C22—C23	1.528 (4)
C3—C4	1.389 (5)	C22—C24	1.530 (3)
C3—H3	0.9400	C22—H22	0.9900
C4—C5	1.376 (4)	C24—C25	1.502 (4)
C4—H4	0.9400	C24—H24A	0.9800
C5—C6	1.395 (4)	C24—H24B	0.9800
C5—H5	0.9400	C25—C26	1.363 (4)
C7—C8	1.520 (4)	C26—H26	0.9400
C7—H7A	0.9800	C27—C28	1.513 (4)
C7—H7B	0.9800	C27—H27A	0.9800
C8—C13	1.386 (4)	C27—H27B	0.9800
C8—C9	1.394 (4)	C29—C30	1.475 (4)
C9—C10	1.390 (4)	C29—H29A	0.9800
C9—H9	0.9400	C29—H29B	0.9800
C10—C11	1.381 (4)	C30—H30A	0.9700
C10—H10	0.9400	C30—H30B	0.9700
C11—C12	1.379 (4)	C30—H30C	0.9700
			
C28—O3—C29	114.8 (3)	C20—C15—C14	121.3 (3)
C21—N1—C1	122.5 (2)	C15—C16—C17	120.7 (3)
C21—N1—C7	117.9 (2)	C15—C16—H16	119.6
C1—N1—C7	119.5 (2)	C17—C16—H16	119.6
C23—N2—C6	123.3 (2)	C18—C17—C16	120.1 (3)
C23—N2—C14	117.2 (2)	C18—C17—H17	120.0
C6—N2—C14	118.5 (2)	C16—C17—H17	120.0
N4—N3—C25	109.2 (2)	C17—C18—C19	120.3 (3)
N3—N4—N5	107.2 (2)	C17—C18—H18	119.9
N4—N5—C26	110.0 (2)	C19—C18—H18	119.9
N4—N5—C27	120.0 (2)	C18—C19—C20	119.5 (3)
C26—N5—C27	129.9 (2)	C18—C19—H19	120.3
C2—C1—C6	119.6 (3)	C20—C19—H19	120.3
C2—C1—N1	118.2 (3)	C15—C20—C19	120.6 (3)
C6—C1—N1	122.1 (2)	C15—C20—H20	119.7
C3—C2—C1	120.5 (3)	C19—C20—H20	119.7
C3—C2—H2	119.7	O1—C21—N1	121.6 (3)
C1—C2—H2	119.7	O1—C21—C22	122.2 (2)
C2—C3—C4	119.8 (3)	N1—C21—C22	116.2 (2)
C2—C3—H3	120.1	C21—C22—C23	105.7 (2)
C4—C3—H3	120.1	C21—C22—C24	111.4 (2)
C5—C4—C3	120.0 (3)	C23—C22—C24	112.6 (2)
C5—C4—H4	120.0	C21—C22—H22	109.0
C3—C4—H4	120.0	C23—C22—H22	109.0
C4—C5—C6	121.1 (3)	C24—C22—H22	109.0
C4—C5—H5	119.5	O2—C23—N2	122.3 (3)
C6—C5—H5	119.5	O2—C23—C22	122.6 (2)
C5—C6—C1	118.9 (3)	N2—C23—C22	115.0 (2)
C5—C6—N2	118.9 (3)	C25—C24—C22	111.2 (2)
C1—C6—N2	122.2 (2)	C25—C24—H24A	109.4
N1—C7—C8	116.9 (2)	C22—C24—H24A	109.4
N1—C7—H7A	108.1	C25—C24—H24B	109.4
C8—C7—H7A	108.1	C22—C24—H24B	109.4
N1—C7—H7B	108.1	H24A—C24—H24B	108.0
C8—C7—H7B	108.1	C26—C25—N3	107.3 (2)
H7A—C7—H7B	107.3	C26—C25—C24	131.7 (2)
C13—C8—C9	118.1 (3)	N3—C25—C24	120.9 (2)
C13—C8—C7	124.2 (3)	N5—C26—C25	106.3 (2)
C9—C8—C7	117.7 (2)	N5—C26—H26	126.8
C10—C9—C8	121.2 (3)	C25—C26—H26	126.8
C10—C9—H9	119.4	N5—C27—C28	111.6 (2)
C8—C9—H9	119.4	N5—C27—H27A	109.3
C11—C10—C9	120.1 (3)	C28—C27—H27A	109.3
C11—C10—H10	119.9	N5—C27—H27B	109.3
C9—C10—H10	119.9	C28—C27—H27B	109.3
C10—C11—C12	119.7 (3)	H27A—C27—H27B	108.0
C10—C11—H11	120.2	O4—C28—O3	125.2 (3)
C12—C11—H11	120.2	O4—C28—C27	125.0 (3)
C11—C12—C13	120.1 (3)	O3—C28—C27	109.7 (3)
C11—C12—H12	120.0	O3—C29—C30	108.4 (3)
C13—C12—H12	120.0	O3—C29—H29A	110.0
C8—C13—C12	120.9 (3)	C30—C29—H29A	110.0
C8—C13—H13	119.6	O3—C29—H29B	110.0
C12—C13—H13	119.6	C30—C29—H29B	110.0
N2—C14—C15	113.5 (2)	H29A—C29—H29B	108.4
N2—C14—H14A	108.9	C29—C30—H30A	109.5
C15—C14—H14A	108.9	C29—C30—H30B	109.5
N2—C14—H14B	108.9	H30A—C30—H30B	109.5
C15—C14—H14B	108.9	C29—C30—H30C	109.5
H14A—C14—H14B	107.7	H30A—C30—H30C	109.5
C16—C15—C20	118.8 (3)	H30B—C30—H30C	109.5
C16—C15—C14	119.9 (3)		
			
C25—N3—N4—N5	0.5 (3)	C15—C16—C17—C18	−0.6 (5)
N3—N4—N5—C26	−0.5 (3)	C16—C17—C18—C19	0.0 (5)
N3—N4—N5—C27	−176.5 (2)	C17—C18—C19—C20	0.4 (5)
C21—N1—C1—C2	−135.6 (3)	C16—C15—C20—C19	−0.3 (4)
C7—N1—C1—C2	48.8 (4)	C14—C15—C20—C19	−177.5 (3)
C21—N1—C1—C6	47.6 (4)	C18—C19—C20—C15	−0.3 (5)
C7—N1—C1—C6	−128.0 (3)	C1—N1—C21—O1	−176.6 (2)
C6—C1—C2—C3	1.4 (5)	C7—N1—C21—O1	−0.9 (4)
N1—C1—C2—C3	−175.5 (3)	C1—N1—C21—C22	0.8 (4)
C1—C2—C3—C4	0.8 (6)	C7—N1—C21—C22	176.5 (2)
C2—C3—C4—C5	−2.5 (6)	O1—C21—C22—C23	101.2 (3)
C3—C4—C5—C6	2.1 (5)	N1—C21—C22—C23	−76.1 (3)
C4—C5—C6—C1	0.0 (4)	O1—C21—C22—C24	−21.4 (3)
C4—C5—C6—N2	178.2 (3)	N1—C21—C22—C24	161.2 (2)
C2—C1—C6—C5	−1.8 (4)	C6—N2—C23—O2	−173.5 (2)
N1—C1—C6—C5	175.0 (2)	C14—N2—C23—O2	−4.5 (4)
C2—C1—C6—N2	−179.8 (3)	C6—N2—C23—C22	9.6 (4)
N1—C1—C6—N2	−3.1 (4)	C14—N2—C23—C22	178.7 (2)
C23—N2—C6—C5	132.3 (3)	C21—C22—C23—O2	−108.4 (3)
C14—N2—C6—C5	−36.6 (4)	C24—C22—C23—O2	13.5 (4)
C23—N2—C6—C1	−49.6 (4)	C21—C22—C23—N2	68.5 (3)
C14—N2—C6—C1	141.4 (3)	C24—C22—C23—N2	−169.7 (2)
C21—N1—C7—C8	77.6 (3)	C21—C22—C24—C25	−80.8 (3)
C1—N1—C7—C8	−106.6 (3)	C23—C22—C24—C25	160.6 (2)
N1—C7—C8—C13	−13.9 (4)	N4—N3—C25—C26	−0.3 (3)
N1—C7—C8—C9	169.2 (2)	N4—N3—C25—C24	−177.0 (2)
C13—C8—C9—C10	0.2 (4)	C22—C24—C25—C26	128.0 (3)
C7—C8—C9—C10	177.3 (3)	C22—C24—C25—N3	−56.2 (3)
C8—C9—C10—C11	−1.4 (5)	N4—N5—C26—C25	0.3 (3)
C9—C10—C11—C12	1.5 (5)	C27—N5—C26—C25	175.8 (3)
C10—C11—C12—C13	−0.4 (5)	N3—C25—C26—N5	0.0 (3)
C9—C8—C13—C12	0.9 (4)	C24—C25—C26—N5	176.2 (3)
C7—C8—C13—C12	−176.0 (3)	N4—N5—C27—C28	93.4 (3)
C11—C12—C13—C8	−0.8 (5)	C26—N5—C27—C28	−81.7 (3)
C23—N2—C14—C15	136.3 (3)	C29—O3—C28—O4	−2.8 (5)
C6—N2—C14—C15	−54.1 (4)	C29—O3—C28—C27	176.7 (3)
N2—C14—C15—C16	139.8 (3)	N5—C27—C28—O4	−9.6 (4)
N2—C14—C15—C20	−43.0 (4)	N5—C27—C28—O3	170.8 (2)
C20—C15—C16—C17	0.7 (4)	C28—O3—C29—C30	−169.4 (3)
C14—C15—C16—C17	178.0 (3)		
